# Supplementary material for: Are carbon emissions trading and green financial instruments synergistic? -Comprehensive quantitative research based on content analysis
Source: PLoS One. 2024 Mar 7;19(3):e0298601. doi: 10.1371/journal.pone.0298601 (PMC10919601; doi:10.1371/journal.pone.0298601)
Supplement: S1 Data — (ZIP) [file pone.0298601.s001.zip › Data/地级市Z_F工作报告.zip!/║╙▒▒/╟╪╗╩╡║╩╨/└┤╫╘╣1⁄2╓┌║┼í╛┬φ┐╦╩2╛▌═°í┐_2014.docx]

　　各位代表：

　　现在，我代表秦皇岛市人民政府向大会作工作报告，请予审议，并请各位政协委员和列席会议的同志提出意见。

　　一、2013年主要工作回顾

　　过去的一年，是全面贯彻落实党的精神的第一年，也是本届政府的开局之年。面对复杂严峻的经济形势，我们在省委、省政府和市委的坚强领导下，围绕建设沿海强市、美丽港城，大力实施开放强市、产业立市、旅游兴市、文化铸市战略，努力打好“五张牌”，深入推进“五大百日会战”，凝心聚力、攻坚克难，经济社会发展实现稳中有进、稳中向好。完成地区生产总值1168.8亿元，比上年增长7%;全部财政收入197.5亿元，公共财政预算收入109.5亿元，分别增长1.3%和0.8%;全社会固定资产投资786.3亿元，增长6.4%;社会消费品零售总额509亿元，增长13.4%;实际利用外资7.5亿美元，增长20.1%，引进内资344.1亿元，增长22.8%;城镇居民人均可支配收入24353元，农民人均纯收入9356元，分别增长10.2%和12.5%;物价保持基本稳定。

　　(一)对内对外开放全面提速。全市人民期盼已久的西港搬迁改造正式实施，作为新一轮开放的最大引擎，被列为省政府一号工程，并得到了国家相关部委的大力支持。西港区、新开河港煤炭运输业务全部关停，相关设施完成保护性拆除。秦皇岛临港产业园区被列入省沿海发展重点支持区域，享受“四税”定额分享、超额全返等政策。把招商引资作为扩大开放重中之重，精心组织“四个入秦专项行动”及赴台专题推介招商等活动，与中粮集团、新兴际华集团、中国节能环保集团、神州数码等220多家央企、知名民企、外企深入对接，签约重大项目56个，中国建材集团新型房屋、联彩石油储备等一批重量级项目取得实质进展。利用外资创改革开放以来最好水平。实施千万元以上在建项目909个，完成投资527.7亿元，中信戴卡汽车零部件、正大食品加工等重点项目顺利推进，哈电核电设备等37个重点项目竣工投产。中关村海淀园全国首家分园落户我市，北戴河新区、海港区申请设立省级高新技术产业开发区获批。完成外贸进出口总额43.7亿美元，其中机电产品出口总额突破12亿美元。

　　(二)产业结构调整不断深化。粮食总产基本稳定，蔬菜、肉、蛋、奶、水产品、果品产量分别达到318万吨、34万吨、11万吨、10万吨、32万吨和87万吨，农业产业化经营率全省领先，达68.5%。着力提升工业经济发展水平，完成工业固定资产投资235.2亿元、增长12%，其中技改投资176.5亿元;新增规模以上工业企业80家，总数达到486家，规模以上工业完成增加值353.3亿元、利润24.4亿元，分别增长2.1%和130%;规模以上工业中装备制造业所占比重达到35.5%，秦皇岛装备制造基地被评为省十大工业基地。大力实施创新驱动战略，10名院士被聘为市政府特邀咨询，清华大学等4所高校将在我市设立研究机构，新增市级以上工程技术中心和重点实验室9家，高新技术企业达到80家，规模以上工业中高新技术产业所占比重达到25%。加快发展现代服务业，完成港口货物吞吐量2.73亿吨，秦港股份在香港上市;全年接待中外游客2595.7万人次，实现旅游总收入256.3亿元，分别增长10.9%和19.6%，旅游业用地改革试点方案获国土资源部批复;世纪港湾等大型商贸项目竣工使用;市商业银行更名为秦皇岛银行，小额贷款公司达到24家、注册资本金达到19.71亿元。民营企业实现增加值761.2亿元，占地区生产总值的65.1%。

　　(三)城乡功能品质明显提升。下大力推进重大基础设施建设，津秦客专建成通车，北戴河机场主体完工，秦皇岛火车站广场、205国道示范线、北戴河机场支线等工程投入使用，民族路上跨铁路桥、文昌路节点改造等社会各界和群众多年关注的重点工程建成通车。投资11.2亿元实施老旧小区和城中村改造项目16个，“我为港城添美丽”等系列活动取得扎实成效。新建绿色节能建筑98.9万平方米，全国首个“被动式——低能耗住宅”示范项目，通过德国能源署和国家住建部验收。实施县城“三治两提”攻坚行动，拆除违法建筑4.3万平方米，四县县城全部成为省级园林县城。深入推进农村面貌改造提升行动，733个重点村全部完成“四清及净化”工作，以望峪村和东西连峪村为代表的30个精品示范点以及2条精品示范线、8个精品示范区面貌显著提升;新改建农村公路430多公里，改造危房1.03万户，解决13.2万农村人口饮水安全问题，治理水土流失65平方公里。我市荣获2012-2013年度河北省人居环境进步奖。

　　(四)生态环境质量持续改善。深入推进北戴河近岸海域环境综合整治三年行动计划，完成投资13.8亿元，新关停和限期治理企业48家，主要入海河流和近岸海域水质达到近年来最好水平。狠抓大气污染综合防治，以壮士断腕的勇气，全面实施大气污染防治行动计划和18个专项治理方案，改造取缔燃煤锅炉135台;淘汰黄标车3.3万辆，当年完成省定三年任务的80%;削减炼铁产能161万吨，淘汰水泥落后产能274万吨。单位生产总值能耗下降3.66%，化学需氧量削减3077吨，二氧化硫、氨氮、氮氧化物分别减排6179吨、208吨和9969吨。启动城乡造林和城区增绿三年行动，全年完成人工造林和封山育林16万亩，森林覆盖率、建成区绿化覆盖率和人均公园绿地面积分别达到44%、50%和20.3平方米。

　　(五)民生和社会事业全面进步。在财力十分紧张的情况下，将公共财政支出的77.5%用于保障和改善民生，认真落实政府为群众办的11件实事。新增城镇就业4.6万人、农村劳动力转移就业1.8万人，城镇登记失业率3.75%。各项社会保险待遇稳步提升，城乡居民社会养老保险在全省率先实现全覆盖。16万低保对象和困难群众基本生活得到保障，1.6万余人实现稳定脱贫。新开工建设各类保障性住房和棚户区改造安置住房1.61万套。农民工随迁子女小学、初中入学率均达99%以上。市中医医院迁入新址，集体产权村卫生室实现全覆盖，新农合参合率达97.13%。先后下调居民用天然气和非居民用天然气价格，下调幅度全省最大。成功承办“国家高新技术产业开发区建设二十年成就展”及秦皇岛科技周活动。我市作为全省第一家国家公共文化服务体系示范区，通过文化部验收。“中国梦·我的梦”主题教育、“圆梦中国·俄罗斯游客北戴河大联欢”等活动达到预期效果。市方志馆成为国家方志馆唯一分馆。圆满举办全国青少年“未来之星”阳光体育大会、第六届市运会等重大赛事，为204个行政村安装了全民健身路径。强化安全生产、食品药品安全监管，深化“双六”工程，扎实推进平安城市建设，社会保持和谐稳定，连续第五次被评为全国社会管理综合治理优秀市。暑期服务保障任务圆满完成。

　　国防动员和双拥共建扎实开展，荣获省双拥模范城“六连冠”。人口计生、民族宗教、广播影视、新闻出版、外事侨务、妇女儿童、防震减灾、地理信息、邮政通信、人防、审计、统计、档案、气象、老龄、慈善、残疾人等事业取得新成绩。

　　(六)政府自身建设进一步加强。认真开展“树讲聚促”和解放思想大讨论活动，有效激发了发展正能量。为更好满足市场主体和人民群众诉求，扎实推进“两转三服务”、“发展环境百日会战”、“提素提质提效”活动，对市本级723项“三类事项”进行清理， 共削减、下放、可改变管理方式506项，保留217项，在全省率先实现市县乡村四级政务服务网络全覆盖、率先开通961890群众服务热线。狠抓“八项规定”、“四项纪律”落实，大力整治“四风”，全面清理和处置违规公务用车、超标办公用房、“吃空饷”人员，市直机关“三公”经费支出比上年压减13%，组织千名社会各界代表对市本级69个系统和单位的工作人员进行“面对面”现场评议，一批损害企业、群众利益的行为得到有效查处。深入推进依法行政工作，修订完善《市政府工作规则》，进一步规范重大行政决策机制和程序。自觉接受人大、政协和社会监督，认真解决代表、委员关注的西港搬迁、发展环境等一批热点问题，累计办理省和市人大代表建议、政协提案516件，按时办复率100%，满意率99%。

　　各位代表!过去一年，我们经受住市场有效需求不足、经济下行压力加大、转型升级任务艰巨等多重严峻考验，取得了来之不易的成绩。这是省委、省政府和市委正确领导的结果，是市人大、市政协和社会各界有效监督、鼎力支持的结果，是全市上下戮力同心、拼搏进取的结果。在此，我代表市政府，向全市人民，向人大代表、政协委员，向各民主党派、工商联、无党派人士和人民团体，向驻秦部队、武警官兵、政法干警和中省属驻秦单位，向所有关心支持秦皇岛发展的港澳台同胞、海外侨胞和国内外朋友，致以崇高的敬意和衷心的感谢!

　　一年来，尽管我们做了大量工作，取得了积极成效，但由于宏观经济形势的影响，加之自身基础相对薄弱等原因，年度目标任务完成情况不尽理想。我们本着实事求是的态度，提请市人大会审议批准，对地区生产总值、全部财政收入、公共财政预算收入、全社会固定资产投资、规模以上工业增加值和社会消费品零售总额6项指标进行了调整，旨在为秦皇岛科学发展、绿色崛起夯实基础、积蓄后劲。我们也清醒地认识到，秦皇岛正处在爬坡过坎、加速转型的特殊时期，前进道路上还面临许多挑战，经济总量小、实力弱的基本市情尚未根本改变，产业结构不优、创新能力不强、大项目支撑不足、投资结构不够合理等矛盾和问题仍旧突出;当前经济运行下行压力依然较大，化解过剩产能任务繁重、部分骨干企业生产经营困难、财政收支矛盾愈加凸显。同时，治理污染和环境保护任务艰巨，社会事业发展和民生改善与群众期望还有差距;转变政府职能和工作作风还不到位，发展环境不够优化，一些部门和工作人员服务意识不强，“吃拿卡要”、“庸懒散”、推诿扯皮等不正之风和消极腐败现象时有发生，纠正“四风”还要狠下功夫、不断深化。对这些困难和问题，我们将进一步强化措施，认真加以解决，确保不断取得实实在在的成效。

　　二、2014年工作安排

　　进入新的一年，虽然宏观经济形势错综复杂，但中央全面深化改革和推进城镇化的重大决策部署，京津冀协同发展战略、河北沿海地区率先发展战略的推进，京津产业的加快辐射外溢，省委、省政府对绿色秦皇岛、活力秦皇岛建设的支持，为我们加快发展提供了有利条件。我们必须咬定目标不放松，抢抓机遇、迎难而上、急流勇进，奋力开创率先开放、赶超发展的新局面。

　　今年政府工作的总体要求是：全面贯彻落实党的、十八届三中全会、中央经济工作会议，省委八届五次、六次全会和市委十一届六次全会精神，深入实施开放强市、产业立市、旅游兴市、文化铸市战略，以“项目建设落实年”、“环境整治攻坚年”、“基层工作服务年”为抓手，着力推进经济结构调整，着力提高发展质量效益，着力加快新型城镇化进程，着力加强生态文明建设，着力保障和改善民生，坚定不移走绿色崛起之路，强动力、增活力、添魅力、壮实力，努力建设全面小康、富裕殷实、山清水秀的沿海强市、美丽港城。

　　主要预期目标是：地区生产总值增长8%;公共财政预算收入增长9%;全社会固定资产投资增长18%;社会消费品零售总额增长13%;进出口总额增长5%，实际利用外资增长10%;单位生产总值能耗下降3.66%，化学需氧量、二氧化硫、氨氮、氮氧化物排放量分别削减1.5%、1.6%、1%和5.5%，细颗粒物(PM2.5)浓度下降5%;城镇居民人均可支配收入和农民人均纯收入分别增长10%以上;居民消费价格指数涨幅控制在3%左右;城镇登记失业率控制在4%以内;人口自然增长率控制在6.3‰以内。需要说明的是，提出这些目标，综合考虑了中央和省的要求以及我市实际，更加注重了经济发展质量和效益。虽然不再以生产总值增长率论英雄，但作为全省沿海发展前沿，我们必须自我加压、负重奋进，努力做到好中求快、既快又好。

　　根据上述目标，我们将紧紧围绕省委、省政府“四大攻坚战”的战略部署、“改革、调整、巩固、提升”的工作主题和绿色秦皇岛、活力秦皇岛建设要求，认真把握好四项原则：一是改革引领，就是牢牢把握全面深化改革这一强大动力，进一步解放思想、解放和发展生产力、解放和增强社会活力，以改革促开放、促发展、促转型、促创新、促惠民，勇当全省深化改革的先行者。二是稳中求进，就是牢牢把握发展第一要务，加快开放步伐，有效发挥“三驾马车”的拉动作用，努力提高经济发展质量效益，做到转变不转向、松绑不松劲、调速不失速，实现稳中有为、稳中提质、稳中增效，确保经济社会发展与日续升。三是创新驱动，就是牢牢把握创新驱动发展核心战略，抢抓新一轮科技革命和产业革命机遇，着力提高自主创新能力、增强核心竞争力，加快建设创新型城市，在全省发挥科技创新引领示范作用。四是科学发展，就是牢牢把握绿色崛起科学内涵，加快优化产业布局、产业结构和岸线利用，增强可持续发展能力，努力以最小的环境代价和最合理的资源消耗，让秦皇岛梦在青山绿水、碧海蓝天下实现。

　　工作中，重点抓好七项主要任务：

　　(一)持续加快率先开放步伐，努力再造一个“开放红利期”。强化开放强市主战略，抓住南资北移、台资北移、外资北移、京津产业外溢等发展机遇，着力打造河北沿海率先发展增长极。

　　一是发挥西港搬迁的引擎作用。统筹处理港产城关系，加快西港搬迁改造步伐。进一步完善东扩港区建设方案，实施港口码头和20万吨航道项目建设，确保年内完成工程量30%以上。着眼培育壮大临港物流园区，加快推进铁路、公路等配套设施以及秦皇岛综合物流信息平台建设，谋划建设商贸物流、保税物流等功能区，打造区域性物流基地。将西港片区项目整体包装，争列国家建设重点。做好西港区土地收储、海域使用权收购以及征收安置补偿工作。按照“先地下后地上”的原则，注重历史文化印迹保护，稳步推进西港区开发改造，争取滨海新城区建设迈出实质性步伐，初步实现还海于城、还海于民。

　　二是狠抓项目建设和招商引资。把今年作为“项目建设落实年”，强化分包负责制，市级抓重大在建和前期项目各100个，县区抓重点在建和前期项目各300个以上;强化要素保障，实行差别化供地政策，发挥政府投资引导作用，确保千万元以上项目投资快速增长，谋划实施100亿元以上项目30个以上。围绕建设先进制造业、战略性新兴产业、现代服务业“三大高地”，持续深化央企、知名民企、外企、引智入秦行动，争取中国建材集团新型房屋、联彩石油储备、中兴恒和北斗产业基地等重大项目落地开工，中信戴卡KSM、正大综合食品等重点项目竣工投产。突出重大基础设施项目的拉动作用，谋划推进承秦铁路、京秦高速二通道、邮轮码头、轨道交通等项目。结合旅游、服务业综合改革试点，放开会计审计、商贸物流、电子商务等领域外资准入限制，推进金融、教育、文化、医疗等领域有序开放。推行产业链招商、精准化招商、常态化驻点招商，主攻京津、长三角、珠三角的同时，强化对台湾、德国等地的招商，借助秦仁航线推动对韩国的招商。今年起，每个县区每年都要争取一定数量的外企落户。

　　三是进一步做强园区载体支撑。落实省提出的开发区产业倍增三年推进计划，对各类园区进行实质性整合，推广“一区多园”模式，促进要素、项目、产业加速集聚。大力推进秦皇岛开发区节能环保及台湾、韩国、欧洲产业园建设，努力打造全省节能环保产业基地，争取设立国家级高新技术产业开发区。加快北戴河新区总部经济、海洋产业等园区建设，抓好动漫旅游城等重大基础设施和重点项目建设，争取离区免税购物等政策，努力创建国家高端医疗健康旅游示范区。充分发挥政策效应，抓好秦皇岛临港产业园区以及北戴河新区、海港区2个省级高新技术产业开发区建设。依托山海关港，对出口加工区、开发区东区及相邻区域实行统一规划，谋划建设综合保税区，培植新的经济增长点。

　　四是加大对接京津的工作力度。强化“八个盯紧”，落实我市与海淀区合作框架协议，以此为突破口，主动承接京津资金、技术、人才、教育辐射外溢，深化在科技创新、产业发展、教育教学、生态保护、城市规划等领域的合作。加强与京津院校、院所、院士的对接合作，抓好清华大学精密仪器和智能装备研究院、北京大学健康产业研究院、北京化工大学海洋化工研究院、天津大学环境工程研究院、国家科技成果展览中心，以及中关村海淀园秦皇岛分园等平台建设，争创省级重大创新基地。推进旅游企业与京津知名旅游公司、旅游品牌的交流合作，组织农业产业化龙头企业与京津地区开展“农超对接”。

　　(二)坚定不移全面深化改革，充分激发经济社会发展内生动力与活力。全面落实市委十一届六次全会《关于学习贯彻党的十八届三中全会和省委八届六次全会精神的决议》，有序推进各项改革，努力实现改革的最大综合效益。

　　一是深化行政管理体制改革。加快政府职能转变，努力为各类市场主体创造统一开放、竞争有序的市场环境。进一步简政放权，承接和落实好国务院、省政府取消和下放的行政审批事项，取消和下放一批市本级行政审批事项;制定并向社会公开行政审批事项目录，没有进入目录的，一律不再审批。稳妥推进市县两级政府机构改革，优化机构设置和职能配置。完善市县乡村四级政务服务体系，扎实开展省级政务服务标准化试点和政府机关标准化管理工作。稳步推进事业单位分类改革，研究探索政府购买公共服务的模式。

　　二是着力激活各类市场主体。坚持权利平等、机会平等、规则平等，着力清除各种市场壁垒，坚决破除“玻璃门”、“弹簧门”。充分发挥市场配置资源的决定性作用，执行负面清单制度，推进投资体制改革，鼓励和引导民间资本进入基础设施、基础产业和公共事业领域。落实鼓励民营经济发展的各项政策，下大力帮扶民营企业“二次创业”，加快打造一批“民企航母”。今年市财政安排的5000万元技改专项资金，其中4000万元用于支持民营企业。推进商事登记制度改革，实行“先照后证”、投资主体零限制、注册登记零收费，放宽注册资本、住所登记条件，激发全民创业热情。大力发展混合所有制经济，积极引进各类资本参与国有企业改制重组、参股国有资本投资项目。改革国有资本授权经营体制，推动国资监管由“管资产”、“管企业”向“管资本”转变。

　　三是加快财政金融体制改革。推进全口径预算编制改革，建立跨年度预算平衡机制，实施全过程预算绩效管理，提升政府财力统筹水平。清理规范重点支出挂钩事项，改革专项资金配套机制，合理划分市县事权和支出责任。落实国家各项结构性减税政策，稳步推进“营改增”工作。推进市级融资平台整合，健全政府债务管理机制，做好化解政府债务风险工作。放宽金融行业市场准入，强化金融市场监管，支持具备条件的民间资本依法设立中小型银行、金融租赁公司和消费金融公司，鼓励民间资本投资入股金融机构和参与金融机构重组改造，争取新设立小额贷款公司5家;支持金融机构到农村增设网点，积极推进村镇银行组建工作。鼓励金融创新，大力培育第三方支付、移动支付及网络金融服务平台等新兴金融业态。做大市级、做实县级担保机构，发挥好17家融资性担保机构作用。大力推动符合条件的企业上市融资，年内每个县区至少实现1家以上企业在各类资本市场挂牌上市。

　　四是稳妥推进农村综合改革。以实现农业强、农村美、农民富为主攻方向，全面深化农村改革，不折不扣落实强农惠农富农政策。在坚持和完善最严格的耕地保护制度前提下，赋予农民对承包地占有、使用、收益及承包经营权抵押、担保权能。建立健全土地流转服务体系，鼓励土地承包经营权在公开市场流转。完善农村宅基地管理制度。探索建立城乡统一的建设用地市场。加大山区综合开发、治理与保护力度，最大限度地激活农村土地资源。积极推动农村集体产权股份制改造，健全完善农村资产评估体系，拓展资产抵押渠道，加快建立县级农村产权流转交易市场。鼓励发展家庭农场，大力发展农民合作组织，年内新发展家庭农场100个、农民合作社100个，农村经济合作组织覆盖率达到80%。培育发展农业公司，引导工商资本到农村发展适合企业化经营的现代种养业和加工业。积极推进农业产业化经营，组建现代农业投资公司，拓宽龙头企业直接融资渠道。

　　五是积极推进社会事业改革。完善财政教育投入稳定增长机制和家庭经济困难学生资助体系，统筹城乡义务教育资源均衡配置。健全市级文化企业国有资产监督管理机制，鼓励非公有制文化企业发展。建立城乡居民大病保险制度和重特大疾病救助机制，推动基本医疗保险制度城乡统筹，全面推开县级公立医院改革。落实一方是独生子女的夫妇可生育两个孩子政策。实施公交、水资源等管理体制机制改革，扩大农业用水终端水价范围。深化基层民主制度建设。

　　(三)深入推进产业转型发展，加快打造秦皇岛发展“升级版”。落实省“三个一百”领军企业工程，一产抓特色、二产优结构、三产提品质，加快构建绿色现代产业体系。

　　一是培育壮大新型工业。落实工业转型升级行动方案和十大提升计划，坚持信息化与工业化相融合，依托中信戴卡、中铁山桥、金海粮油、耀华集团等骨干企业，开展好“对标”行动，实施七大技术改造专项和百项技术改造工程，努力建设全省最重要的临港重大装备制造基地，促进粮油食品、金属压延、玻璃工业向产业链和价值链高端攀升。加快发展高新技术和战略性新兴产业，年内争取滚动实施高新技术研发和产业化项目70个。深化与中国节能环保、神州数码、中国医药等集团的合作，培育壮大节能环保、数据产业、生物医药、新材料、新能源等产业。实施海洋产业振兴工程，积极发展海洋经济。深入推进中小企业成长工程，促进“个转企、小升规”，助推星箭特种玻璃、思泰意达、领先科技、3D打印、中科遥感等一批高成长性科技“小巨人”快速成长，年内新增规模以上工业企业70家左右、主营业务收入超亿元企业20家以上、高新技术企业8家，科技型中小企业达到550家。搞好工业运行分析，加强困难企业帮扶，力促工业经济提速提效。

　　二是大力发展现代服务业。强化旅游兴市战略，促进旅游与文化的融合，加快旅游业转型升级，推进国际旅游度假中心、葡萄岛、五佛山森林公园、石河南岛等中高端项目建设，开展好第三届中俄国际旅游交易会等活动。大力发展物流业，推进国家级煤炭交易中心、国际航运服务中心、区域性农产品交易市场和专业市场建设，实行商检、海关、金融、保险、代理等“一站式”服务，推进口岸大通关。积极发展文化创意产业，加快山海关长城文化产业园、北戴河怪楼文化艺术产业园、艺术品交易中心等项目建设，培育知名文化品牌。吸引国内外知名康体医疗机构在我市设立分支机构，发展康复养老、医疗器械、体育服务等产业，打造“健康秦皇岛”品牌。加强与国经中心的战略合作，推进光大国际金融商务港、银亿国际服务外包科技产业园等项目，壮大现代金融、服务外包、总部经济等新兴服务业态。关注房地产市场动态，研究针对性政策措施，促进房地产业健康发展。积极扩大消费需求，培育休闲度假、文化教育、家政服务等新兴消费模式，推进茂业城市综合体等项目以及农贸市场、县城商贸流通设施建设，支持“万村千乡”连锁配送企业发展;抓好国家信息消费城市建设试点工作，落实省电子商务三年发展推进计划，努力扩大电子商务交易和信息消费规模;实施城乡居民收入倍增计划，认真落实鼓励居民消费各项政策，提高第三产业贡献率。

　　三是积极发展现代农业。全力推进现代农业“双五双十双百”行动，促进农业增效、农民增收，加快走出一条沿海新型农业发展之路。着力加强农业规模化、标准化基地建设，确保农产品质量安全。稳定粮油播种面积，建设粮油标准化生产基地200万亩。大力发展蔬菜和林果产业，新增设施蔬菜种植面积1万亩、优质果品栽培面积3万亩。适度扩大畜牧和海洋水产养殖规模。围绕农产品加工、农产品物流、生态循环农业、国家级现代农业和山区综合开发五类园区建设，加强项目包装和招商工作，延伸农产品产加销链条，拓展农业的多功能性，农业产业化经营率较上年提高1.5个百分点，打造农产品加工强市。建立健全“三农”综合信息服务平台，深入实施优种工程，发展品牌农业，新增一批无公害、绿色、有机农产品。以吸引年轻人务农、培育职业农民为重点，扎实推进农村劳动力培训阳光工程，培育一批职业农民。

　　四是健全科技创新体系。结合国家创新型城市试点工作，加强政产学研相结合的自主创新体系建设。建立科技创新市场导向机制，谋划建设技术转移交易中心和科技成果孵化基地，支持科研人员领办、创办科技型企业，引导企业加大研发设计投入，促进科研成果资本化、产业化。加强院士工作站建设，搭建一批具有国内外领先水平的工程化平台和技术服务平台，发展一批企业主导、产学研用紧密结合的产业技术创新联盟，年内新建技术研发机构10家，争取2家进入省级序列。落实人才有关政策，大力培养、引进各类拔尖人才、创新人才和领军人物。

　　(四)积极稳妥推进新型城镇化，提高城乡一体化发展水平。抓住省统筹城乡一体化发展试点市机遇，落实省委“十要十不要”的要求，努力探索具有自身特色的城乡统筹协调发展之路。

　　一是增强城市区的辐射带动能力。按照西移北进、保护生态、组团发展的思路，传承历史文脉，有序拓展城市空间，完善“4+2”城镇组团布局，一张蓝图干到底，让城市融入大自然，让居民望得见山、看得见水、记得住乡愁。加强城市基础设施配套建设，力争北戴河机场暑期正式通航，确保北环路、韩石路上跨铁路桥建成通车;谋划实施公路新改建工程8项130.6公里，推进沿海高速北戴河联络线、兴凯湖路南延伸、京哈高速祖山连接线等工程，着力打造市域“半小时交通圈”。持续加大城市老城区、城乡结合部整体改造力度，推进火车站周边片区、海阳镇、北戴河北部新城、山海关石河周边等重点区域开发建设。加强城市地下空间利用和地下综合管廊建设，加快海港区北部片区等3个污水处理厂和北戴河西部水厂建设，抓好城市区主要街道两侧、重点区域、关键节点的美化、亮化，提升城市功能品质和吸引力。

　　二是加快县城建设和县域经济发展。实施“大县城”战略，按照一县一特色原则，抓好县城控制性详细规划、各类专项规划编制完善工作。推进昌黎、抚宁县城与主城区同城化发展，打造富有浓郁滨海特色的卫星城;推进青龙、卢龙县城建设彰显历史文化元素、民俗风貌、旅游特色的中小城市。深入开展“三治两提”攻坚行动，搞好水、电、路以及垃圾、污水处理等设施建设，力促县城环境容貌改观和管理水平有效提升。加强小城镇建设，在各县区挑选1-2个建制镇重点支持，努力培育形成一批产业重镇、商贸强镇、旅游名镇和综合型小城镇。搞好小城镇环境治理，大力治脏、治乱，打造一批环境优美小城镇。落实省县域经济发展“1+3”政策措施，将资金、土地等要素向县域倾斜，推动县域园区功能整合，促进产业向园区聚集、园区向县城集中，加快抚宁际华军民产业园、昌黎华夏七期扩建、卢龙英利集团光伏发电等重点项目建设，进一步做优做强葡萄酒等优势产业，发展壮大新型工业。各县要以跻身全省县域经济30强为目标，有选择、有侧重地培育1-2个特色主导产业，加快做大经济总量。

　　三是进一步理顺城镇管理体制机制。有序放开城市区落户限制，放开小城镇和县城落户限制。全面实行流动人口居住证制度，逐步推进居住证持有人享有与居住地居民相同的基本公共服务。抓好智慧城市试点工作，大力实施智慧旅游、智慧医疗、智慧交通、智慧环保、智慧安监、智慧民生等项目，在全市域推行数字化城管，着力解决交通拥堵、环境恶化等“城市病”，加强物业管理行业监管，提高城镇管理精细化、网格化、法制化水平。树立城镇资源商品化、建设产业化、管理企业化理念，强化政府主导地位，完善市场机制，搞好土地一级开发，盘活存量资产;整合市级国有城建投融资平台，鼓励有条件的县设立新型城镇化建设投资集团，引导社会资本参与城市经营、建设和管理。

　　四是切实促进城乡一体化协调发展。坚持工业反哺农业、城市支持农村和多予少取放活方针，促进城乡共同繁荣发展。实施扶贫开发“1234”工程和“五位一体”扶贫开发模式，加大产业、金融扶贫力度，通过山区农业综合开发、股份合作、家庭手工业等方式发展脱贫增收项目，确保2.8万贫困对象稳定脱贫。加快青龙满族自治县脱贫步伐。扎实推进美丽乡村建设，抓好农村面貌改造提升行动，高标准完成136个村庄的改造提升任务，改造农村危房1万户以上。加强农田水利建设，发展节水灌溉面积4万亩，新建水池水窖1000个，治理水土流失65平方公里，解决11万人饮水安全问题。推进城乡基本公共服务均等化，加快水电路气讯等设施城乡联网、共建共享，制定实施城乡统一的教育、卫生、养老等设施配置和建设标准。把北戴河区、秦皇岛开发区率先建设成为全市统筹城乡一体化发展的示范区。

　　(五)着力加强环境治理和生态保护，加快打造生态文明先行区。落实“环境整治攻坚年”，在加强软环境建设的同时，进一步加大硬环境投入和整治力度，努力建设宜居宜游、生态低碳的滨海绿色之城。

　　一是坚决打好大气污染防治攻坚战。严格落实国家、省各项部署要求和市18个专项实施方案，以零容忍的态度，强力抓好大气污染防治工程。下决心治理抚宁石门寨镇、昌黎朱各庄镇、卢龙石门镇等重点区域，确保违法违规排污企业关闭整改到位。坚决推进钢铁、水泥、玻璃等行业大气污染治理攻坚行动，实施工业企业脱硫脱硝项目14个、除尘项目36个，取缔燃煤锅炉468台，完成港口翻车机干雾抑尘改造和堆煤场洒水除尘升级改造，推进集中供热、“煤改气”、锅炉改造工程建设。减少机动车污染排放，推广新能源汽车，优先发展公共交通，争取两年内在全省率先完成黄标车淘汰任务。严格控制扬尘污染，大力治理餐饮业排污，实施农作物秸秆压块替代燃煤工程，全面禁止秸秆焚烧。进一步完善重污染天气应急响应机制。

　　二是持续治理好近岸海域环境。实施北戴河近岸海域环境综合整治第三个年度行动计划，优化工作方案、强化责任落实，切实抓好总投资56.5亿元的67个治理项目。重点加强石河、沙河等入海河流和青龙河综合整治，强化工业点源污染、农业面源污染治理，加大违法违规企业关停和排污企业监管力度，促进海水质量持续好转。进一步落实河流保洁和环境卫生“河长制”、“片长制”等制度，健全海域治理长效机制和流域水环境生态补偿机制。加大水源地环境监察和保护力度，努力让人民群众喝上干净水、放心水、安全水。落实海洋渔业资源保护制度，不断改善海洋生态环境，坚决打击“绝户网”，促进海洋渔业资源恢复。

　　三是强力抓好节能减排。建立健全企业能耗管理体系，实施百家企业重点节能工程，确保累计净压减炼铁产能251万吨以上。支持符合产业政策的企业开展区域兼并重组、技术改造等结构调整工作。提高企业环保、节能、技术准入门槛，严格执行能耗限额、污染物排放特别限制标准和差别电价、水价政策，倒逼不达标产能退出市场。实施效能提升工程，以国控重点企业为龙头，统筹推进结构、工程和管理减排。加快建立区域污染物总量控制指标体系，对污染严重县区实行污染物特别排放限值、建设项目排放总量指标及产业项目区域限批。做好低碳城市试点工作，广泛开展节地、节水、节电、节材活动，大力发展循环经济和清洁生产，加快北戴河新区绿色节能建筑示范区建设，城市区所有新建建筑均按照绿色建筑一星级以上标准建设管理。

　　四是扎实推进生态环境保护建设。严格落实生态功能区规划，划定生态保护红线，加强湿地、土壤环境保护和综合治理，坚决控制地下水超采。落实城乡造林和城区增绿三年行动计划，以河流沿线和高速公路、沿海公路两侧及生态脆弱区等部位为重点，不断提高城乡绿化规模和质量;实施山水林湖生态工程，抓好北戴河湿地及周边生态修复、公园绿地建设改造、暑期线路景观和小区街巷增绿等重点园林绿化项目，确保城区新增绿地41万平方米、人工造林9.1万亩，森林覆盖率、建成区绿化覆盖率、人均公园绿地面积分别达到44.5%、50.45%和20.49平方米。深入推进城乡环境卫生综合治理，加大高速公路、铁路沿线、景区周边等重点区域环境整治提升力度，确保暑期前城市容貌有新的改善。强化执法监管，构建政府主导、环保监管、部门协同、企业落实、社会参与的环境治理机制，建立健全市县乡村四级网格化监管体系，从严打击环境违法犯罪行为。

　　(六)抓住保障和改善民生这个根本，推动社会事业持续健康发展。坚持保障民生投入、安排民生项目、解决民生问题三个先行，认真办好民生实事，今年市本级公共财政支出预算用于民生的支出比例达到80%以上，让发展成果更多更公平惠及全市人民。

　　一是推动实现更高质量就业。完善政府投资项目与增强就业联动机制，多渠道创造就业岗位;深入实施“创业帮扶工程”，规范小额贷款担保基金使用管理，提升市、县区创业指导中心服务水平，努力让劳动者就业有岗位、创业有门路。实施离校未就业高校毕业生就业促进计划，完善中小微企业吸纳高校毕业生就业扶持政策，确保应届毕业生就业率达到85%以上。全面落实退役士兵安置改革政策。加大基层就业服务平台建设力度，强化各类技能培训和职业指导，统筹做好农村转移劳动力、城镇就业困难人员等重点群体就业工作，高度重视化解过剩产能出现的下岗人员再就业工作，努力解除他们的后顾之忧。加大劳动监察执法力度，有效保障劳动者合法权益。年内新增城镇就业4.5万人，农村劳动力转移就业1.8万人。

　　二是提高社会保障水平。完善最低工资和工资支付保障制度，规范收入分配秩序，增加低收入者收入，扩大中等收入者比重。进一步推进非公有制企业、个体工商户、进城务工和灵活就业人员社会保险扩面，逐步推进社会保险标准化建设，积极推进城镇职工基本医疗保险市级统筹。提高企业退休人员基本养老金和城乡居民社会养老保险基础养老金水平。完善城乡低保政策，城乡低保标准分别达到全年5400元和2500元，城乡居民医疗补助标准人均提高到320元。提高优抚对象待遇标准，健全社会救助和保障标准与物价上涨挂钩联动机制，努力把保障困难群众基本生活的底线兜住兜好。推进城乡一体化养老服务体系建设，扩大城市居家养老服务中心和农村互助幸福院覆盖范围，探索实施政府购买养老服务新模式，为老年人安享晚年创造更好的条件，让子女们安心、放心。落实“四房并轨”运行，新开工保障性住房2250套，改造棚户区住房8750套，帮助更多的城乡群众圆好“安居梦”。

　　三是协调发展各项社会事业。办好人民满意的教育，加快义务教育均衡发展步伐，保障进城务工人员随迁子女平等接受义务教育，让他们不再为上学问题发愁;继续推动学前教育发展，实施普通高中教育特色创建工程，支持秦皇岛职业技术学院等国家骨干高职院校建设，重视特殊教育工作，促进高等教育特色学科建设，提高校长、教师两支队伍素质。加强城市社区卫生服务中心建设，抓好县级中医院基础设施建设，实施基层中医药服务能力提升工程;政府办基层医疗机构全部实施基本药物制度、确保网上采购率保持100%;推进市第二医院、军工医院迁建工程和市精神卫生中心建设，谋划实施公立医院整合重组工作，鼓励社会资本举办医疗机构，为群众提供更多的医疗资源、创造更好的就医条件。关爱残疾人，建立贫困重度残疾人生活补贴和贫困残疾儿童抢救性康复补贴制度。推进市科技馆、“云科普”服务平台建设，提升市民科学素质。巩固公共文化服务体系示范区创建成果， 深入实施公共文化服务设施提档、惠民提质、服务提效工程;加快市博物馆、美术馆等文化设施建设，推进数字文化服务，实现文化共享工程村级服务点全覆盖，构建“33111”文化设施网络。深入推进全民健身工程，完成100个行政村全民健身路径的安装。

　　四是维护社会安全稳定。实施“打非治违”、非煤矿山、尾矿库、管道管网、危险化学品等专项治理，持续开展安全生产大检查活动，加强重点行业领域安全监管和作业场所职业危害监管，坚决遏制重特大事故发生。深入开展食品药品隐患排查和“三打”专项行动，实施食品药品安全示范县区创建工作，建立健全市级农产品质量安全追溯系统和产出地准出机制，确保人民群众“舌尖”上的安全。加强突发公共事件应急预警，完善社会稳定风险评估机制。扎实做好信访工作，强化法律在化解矛盾中的权威地位， 有效维护社会公平正义。切实抓好平安城市建设，加强社会综合治理，深入推进农村社会管理“双六”工程，推行街道社区网格化管理模式，建立多元化调节民事纠纷机制，依法严厉打击违法犯罪行为，确保社会和谐稳定。 坚持服务与发展双线并举、互促互进，扎实做好服务暑期和借力暑期工作，确保实现安全、和谐、简朴、满意的暑期目标。

　　加强国防教育和国防后备力量建设，深入开展双拥共建活动，推进军民融合深度发展。支持工会、共青团、妇联、科协等人民团体发挥桥梁纽带作用，做好新闻出版、广播电视、老龄、妇女儿童、气象、防震减灾、地理信息、民族宗教、外事侨务、人民防空、邮政通信、审计、统计、方志、档案和援疆援藏等工作，推进各项事业取得新进步。

　　(七)加强政府自身建设，提升人民群众对政府工作的满意度。以党的群众路线教育实践活动为动力，把今年作为“基层工作服务年”，扎实推进政府科学化、法治化、制度化、标准化建设，努力提高政府的公信力、执行力和服务力。

　　一是不断增强依法行政、科学决策的能力。强化法治思维和法治意识，严格按照法定权限和程序行使权力，依法公开权力运行流程，保证权力正确行使。巩固政府规范性文件清理成果，规范行政裁量权，全面落实行政执法责任制，加强行政复议规范化建设，切实做到有法必依、执法必严、违法必究。认真执行民主集中制，坚持科学民主决策，最大限度地规避风险和失误。全面落实人大及其会决议决定，主动接受政协民主监督，广泛听取社会各界的意见建议，重视发挥新闻舆论和人民群众监督作用。认真办理人大代表建议和政协提案。深化政府信息公开，畅通政府与群众双向沟通渠道，提高政府工作透明度。

　　二是始终保持奋发有为、务实高效的作风。牢记“两个务必”，坚定理想信念，提振“精气神”，练就“真本事”，锻造“好作风”，务实重行，敢于担当，树形象、聚民心、增合力。深化“两转三服务”和“提素提质提效”活动，千方百计为群众、基层、企业办实事、解难事。持续推动思想大解放，切实打破惯性思维束缚，努力以改革的思路、创新的方法破难题、解瓶颈。建立健全责任落实机制，制定“路线图”和“时间表”， 充分运用民主评议这一有效载体，持之以恒抓落实，做到有部署、有检查、有结果、有奖惩。落实“八项规定”，坚决抵制“四风”，用好批评和自我批评有力武器，认真查找和医治行政机关肌体中的顽症，从严治理“假、浮、庸、懒、散、奢”等问题。大力精文简会，切实改进文风会风。

　　三是坚决落实从严治政、廉洁从政的要求。认真落实党政机关厉行节约反对浪费条例，从严控制“三公”经费和一般性支出，坚决遏制公务支出和公款消费中的各种违规违纪违法现象，市直机关“三公经费”支出比上年压减15%以上。认真落实中央《建立健全惩治和预防腐败体系2013-2017年工作规划》，将防腐举措与各项工作同步谋划、同步部署、同步实施，完善提升“体制机制+科技+文化”防治腐败模式，推动电子监察平台与公共资源交易平台有机衔接，建立廉政风险动态管理机制，以零容忍态度严厉查处违法违纪案件，绝不养痈遗患。全面履行党风廉政建设责任制，严格执行廉洁从政各项规定，做到严格自律、克己奉公。

　　各位代表!万马奔腾启新程，扬鞭追梦一路春。新一轮改革开放的号角催人奋进，新阶段绿色崛起的使命艰巨光荣。让我们紧密团结在以同志为的党中央周围，在省委、省政府和市委的坚强领导下，解放思想、开拓创新、同频共振、奋发进取，共同谱写建设沿海强市、美丽港城的新篇章!
